# Confessions of an educational psychologist

**DOI:** 10.3389/fpsyg.2014.00892

**Published:** 2014-08-21

**Authors:** Kim J. Calder Stegemann

**Affiliations:** Faculty of Human, Social and Educational Development (School of Education), Thompson Rivers UniversityKamloops, BC, Canada

**Keywords:** teaching educational psychology, initial teacher education, twenty-first century

I am always attempting to develop new and better ways to teach beginning teachers “all there is to know” about child development and special education. Over the years this process has lead me to feel more and more disillusioned. I am convinced that we need a total revamp of how we approach teaching educational psychology and propose that our discipline re-define what is considered to be essential knowledge and skills for teachers in the twenty-first century. I begin my argument with a review of typical educational psychology courses in initial teacher education, and what my experience with content and process has been over the past 19 years of my career. I then outline the basic tenets of twenty-first century learning and compare these to a “typical” introductory course in educational psychology. Finally, I suggest possible alternatives to the current curriculum and pedagogy of educational psychology in order for it to align with the principles of twenty-first century learning.

## Typical introductory course in educational psychology

### The content

In Canada, most Bachelor of Education programs offer an introductory course in child development, typically over one semester of the program. The semesters tend to be 13 weeks in duration, with a minimum of 36–39 contact hours for three course credits. In the 19 years that I have been teaching the introductory child development course, I have never ended the course saying, “Wow, I ran out of things to teach.” In fact, I usually end up cutting out content, having responded to the needs of my teacher candidates (TCs). For example, though I schedule several weeks for the TCs to digest basic statistics and research methods in order to critically review research articles, some TCs inevitably struggle with the concepts and require more time to synthesize the content. On other occasions I have made adjustments to the pace and content of the course, based on some field experiences that have been discussed in class. But never have I finished my courses and felt that I covered everything that I believed was important, outlined in the textbook, or even listed on the course outline, for that matter.

What are the common topics that we usually include in our child development courses? Theories of development and learning, of course! Have any of us ever taught the course without ensuring that Piaget was covered? Or Vygotsky? Or Gardner? Those seem to be among the “biggies” to introduce. I consider myself lucky if we ever get to theories of motivation, metacognition, and cognitive processes. In addition to the course outline and content, we are also required by our department to infuse cross-cultural awareness, technology, and classroom management. The plate of topics and content is enormous!

Friesen and Jardine (2010) echo this sense of “too much to cover in too little time” in their discussion of twenty-first century learning. They note that schools are “… accelerating, continually differentiating and multiplying the tasks that are asked of them, while, at the same time, attempting to leave in place the structures and practices that were responsive and responsible ventures over one hundred years ago” (p. 5). The same can be said of post-secondary education. Though teacher educators espouse “best practice”—teaching relevant curriculum in meaningful ways that engage all learners—we aren't practicing what we are preaching. There seems to be too much content, compressed into too little time, and this leads instructors and students to revert to the “memorize and regurgitate” pattern of teaching and learning.

### The processes

I have tried to relieve my stress and discomfort about omitting content by devising assignments that help to synthesize and apply the material in meaningful ways. For example, most recently this has taken the form of analyzing TCs' practicum lesson plans so that they will better understand “why they are doing, what they are doing.” Despite these efforts to connect theory to practice, and to make the course “relevant,” I can't help but feel that the courses have been marathons and the students and I just runners in the pack, trying to get finished. “Check. Another course down,” says the TC.

Yet, the purposes of having educational psychology situated within teacher education programs is so that teachers can better understand the learning process, and to ultimately improve education (Berliner, 2012). The current curriculum and pedagogy of introductory educational psychology courses, however, put our discipline in jeopardy of being deemed irrelevant for pre- or in-service teachers.

### The bigger question

Every year I change the assignments, resources, and daily lesson plans to help TCs develop essential understandings. Every year I wonder (and would encourage others to consider): *What do teacher candidates really need to know in order to make beginning teaching a successful experience and as a foundation for later practice?*

## Re-focusing introductory educational psychology courses

While the contributions of legendary educational psychologists have enriched our discipline and served to improve teaching practice, we must look for new ways to keep our discipline relevant to modern-day classrooms and instruction. As Berliner and Calfee state in the first Berliner and Calfee (2004), our discipline changed substantially from twentieth century pre-war eras, and it will no doubt look significantly different in the years to come. I am suggesting that one possible way to realign our practice is by drawing upon the directives for twenty-first century learning. Every province and territory in Canada has position papers which outline the direction of public education for the new millennium (Boudreault et al., 2012/2013; C21 Canada, 2012; Dunleavy et al., 2012). Internationally, there is also a push to critically examine public education to better meet the needs of learners in a world that is vastly different from the nineteenth or twentieth centuries (Ananiadou and Claro, 2009; OECD, 2010; Partnership for 21st Century Skills, 2011; Schleicher, 2012). Because of this widespread adoption and focus, I am proposing that we consider revamping our courses to align with these initiatives.

### Principles of twenty-first century learning

There are numerous frameworks for twenty-first century learning (see Dede, 2009 for a comparative analysis). As a sample, I am referring to two western provinces in Canada (British Columbia and Alberta) which have outlined the principles of twenty-first century learning by listing the skills and dispositions that citizens will need in order to thrive in a knowledge-based society: (1) collaboration and leadership, (2) critical thinking and problem solving, (3) creativity and innovation, (4) social responsibility; cultural, global, and environmental awareness communication, (5) digital literacy, and (6) lifelong learning, self-direction, personal management (adapted from Premier's Technology Council, 2010; Alberta Education, 2011).

These documents further identify how the education system must change. Our education systems must move away from: (a) learning information to learning to learn, (b) data consumption to discovery, because content is changing rapidly, (c) “one-size-fits-all” to tailored learning (project-based), (d) testing to assess to assessing to learn, (e) classroom to lifelong learning and learning for authentic purposes, (f) teacher as lecturer to teacher as guide, and (g) passive to active learners (adapted from Premier's Technology Council, 2010; Alberta Education, 2011). Others have framed these changes in terms of: (a) Ways of Thinking (creative, innovative, critical, decision-making, learning to learn/metacognitive), (b) Ways of Working (communication, collaboration), and (c) Tools for Working (information literacy, ICT literacy) (Binkley et al., 2012).

Nowhere in any of these visions of education does it focus on just acquiring knowledge such as theories, theorems, or basic facts. Rather, the focus is on *thinking* and *working* differently, which is a challenge for introductory educational psychology courses, which continue to be theory-laden.

#### “I'll make it fit”

As you review the first list above, you could argue that you can do all of those things within your current educational psychology courses. For example, you might say that your TCs already work collaboratively on projects that you assign, that they must communicate effectively with peers, and that they think critically and solve problems or questions that you pose. Furthermore, you may say, as I have, that TCs must already be self-directed learners to be admitted into a teacher education program. They must already be concerned citizens who want to make the world a better place, particularly for children. I could stop right there and not really change anything in the way that I teach my introductory educational psychology courses, but there is more to consider.

#### Doing it differently

The list on the teaching and learning process is at the heart of change in the twenty-first century framework. This is where I am really challenged, beginning with the very first tenet—from learning information to learning to learn. Does it mean that theory is no longer the driving force for courses in child development? Maybe, rather than being consumers of knowledge, my TCs need to be discoverers of knowledge (tenet #2). What if our TCs develop their own theories of why and how children and youth develop and learn? Now we are getting somewhere!

Tenet #3 talks about personalized learning. So, if one-size curriculum doesn't “fit all,” maybe that means that I need to allow my students to pose the problems and create their own projects that are relevant to their assigned practica. Maybe it means that the course learning outcomes have only suggested topics. For example, maybe some TCs will explore language development because they are placed in classrooms of multilingual learners, while other TCs will focus on motivational theory because they are particularly challenged by students who are turned-off school.

When I allow my TCs to determine their own projects, I am fostering learning for authentic purposes (tenet #5), and facilitating self-directed learning. I am developing the “habits of the mind,” which I hope will carry forward into their future careers. This also relates to tenet six. If I support my TC's self-selected projects, I am surely being a guide and no longer the “sage on the stage.” In the end, this helps me to achieve the seventh tenet—that my students are active participants and not passive receptacles.

My introductory educational psychology course will look distinctly different now. I have changed the way that I teach my course and the way that my TCs are “thinking” (Binkley et al., 2012). But I still wonder how else I need to re-structure the course to promote new “ways of working”? One option might be to create professional learning communities (PLC), which would include both TCs and practicing teachers. The purpose of the PLCs is to promote collaborative problem solving and action research. TCs could help analyze situations and solve problems that are most pressing for classroom teachers. By doing this, I establish the expectation that teachers continually reflect on their practice, make adjustments, and try new strategies or approaches. It also reinforces the notion that collective examination and conversation can be highly effective to improve educational practice.

I could go on believing that what I am currently doing can “fit” into the principles of twenty-first century learning. A much better fit is to look at the discipline “anew.” The first step is accepting that our discipline must evolve in order to remain relevant for teaching and learning in current and future educational contents. That does not mean “tossing the baby out with the bath water,” but it should spur us to critically examine how our discipline can continue to make significant contributions to education. Utilizing the principles from the twenty-first century learning literature is only one way to reframe; I am sure that there are many others. I want to begin the dialog.

“We need to rethink how to transform public education to ensure relevancy for today's modern learner” (C21 Canada, 2012, p. 4).

### Conflict of interest statement

The author declares that the research was conducted in the absence of any commercial or financial relationships that could be construed as a potential conflict of interest.
